# Struggling to resume childhood vaccination during war in Myanmar: evaluation of a pilot program

**DOI:** 10.1186/s12939-024-02165-9

**Published:** 2024-06-13

**Authors:** April Poe, Emily, Aurora, Hein Thura Aung, Alfred Saw Ei Reh, Brianna Grissom, Cynthie Tinoo, Daniel B. Fishbein

**Affiliations:** 1 Ethnic Health Professional Association, Naypyidaw, Myanmar; 2 Expanded Program on Immunization, Ministry of Health and Education, National Unity Government, Naypyidaw, Myanmar; 3 Department of Statistics & Applied Probability, University of California - Santa Barbara, Santa Barbara, CA USA; 4 Burmese Medical Association of North America, Baltimore, MD USA; 5 Independent Researcher, Santa Barbara, CA USA

**Keywords:** Vaccination, Myanmar, Infant, Ethnic and racial minorities, Parents, Developing countries, Retrospective studies, World health organization, Nurses role, United nations, International cooperation

## Abstract

**Background:**

After the military coup in Myanmar in February 2021, the health system began to disintegrate when staff who called for the restoration of the democratic government resigned and fled to states controlled by ethnic minorities. The military retaliated by blocking the shipment of humanitarian aid, including vaccines, and attacked the ethnic states. After two years without vaccines for their children, parents urged a nurse-led civil society organization in an ethnic state to find a way to resume vaccination. The nurses developed a vaccination program, which we evaluated.

**Methods:**

A retrospective cohort study and participatory evaluation were conducted. We interviewed the healthcare workers about vaccine acquisition, transportation, and administration and assessed compliance with WHO-recommended practices. We analyzed the participating children’s characteristics. We calculated the proportion of children vaccinated before and after the program. We calculated the probability children would become up-to-date after the program using inverse survival.

**Results:**

Since United Nations agencies could not assist, private donations were raised to purchase, smuggle into Myanmar, and administer five vaccines. Cold chain standards were maintained. Compliance with other WHO-recommended vaccination practices was 74%. Of the 184 participating children, 145 (79%, median age five months [IQR 6.5]) were previously unvaccinated, and 71 (41%) were internally displaced. During five monthly sessions, the probability that age-eligible zero-dose children would receive the recommended number of doses of MMR was 92% (95% confidence interval [CI] 83–100%), Penta 87% (95% CI 80%–94%); BCG 76% (95% CI 69%–83%); and OPV 68% (95% CI 59%–78%). Migration of internally displaced children and stockouts of vaccines were the primary factors responsible for decreased coverage.

**Conclusions:**

This is the first study to describe the situation, barriers, and outcomes of a childhood vaccination program in one of the many conflict-affected states since the coup in Myanmar. Even though the proportion of previously unvaccinated children was large, the program was successful. While the target population was necessarily small, the program’s success led to a donor-funded expansion to 2,000 children. Without renewed efforts, the proportion of unvaccinated children in other parts of Myanmar will approach 100%.

**Supplementary Information:**

The online version contains supplementary material available at 10.1186/s12939-024-02165-9.

## Background

Following the election of its first democratically elected government in 2015, Myanmar, the largest country in Southeast Asia, had a period of hope, relative peace, and progress [1–3] compared to the oppression under previous military governments [4]. Progress in the health sector [3] was exemplified by the Expanded Program on Immunization (EPI), under which the immunization coverage exceeded that of other low-middle-income countries [5]. However, inequities, particularly in poor and ethnic minority communities, tarnished this progress [6].

After the military coup in February 2021 [4, 7–10], the health system began to disintegrate [8, 10–15] when senior medical leaders were persecuted for opposing the coup, and medical staff at all levels left their now military junta-controlled jobs and joined the Civil Disobedience Movement [16–18]. The EPI collapsed when its director was imprisoned, and other staff fled [16, 18, 19]. The proportion of children who received their third dose of diphtheria-containing vaccine declined rapidly, falling from 84% in 2020 [20] to 37% in 2021 [20]. Fears of outbreaks of vaccine-preventable diseases followed [21].

The military junta attempted to eliminate dissidence by blocking the shipment of humanitarian aid, including vaccines, and attacking civilian targets [22], including health facilities and staff [8, 18]. The consequences were particularly profound in states governed by ethnic minorities, where deteriorating physical and mental health followed, especially among internally displaced persons (IDPs) [23–26]. Despite the ongoing conflict, many Civil Disobedience Movement health workers and civilians fled to such states because they were governed semi-independently by ethnic minorities [14, 23, 27].

In one state, a nurse-led civil society ethnic health organization was urged by parents to provide vaccines to their children. They formed a Vaccination Team and collaborated with a Civil Disobedience Movement member who was previously an immunization expert with the precoup government and was now in exile, serving as a member of the National Unity Government [28, 29]. Although United Nations agencies could not provide vaccines or financial support [30], saying it was first necessary to conduct a pilot program to demonstrate that the resumption of vaccination was feasible. We report a description and evaluation of such a pilot program and assess the feasibility of its expansion.

## Methods

### Aim

Describe and conduct a participatory evaluation of a pilot program trying to resume childhood vaccination and assess the feasibility of its expansion.

### Setting

An ethnic state of Myanmar where internally displaced people fled after their communities were destroyed or after they left government-controlled jobs.

### Participants

184 children and the vaccination team.

### Design

From July through October 2023, we conducted open-ended interviews and obtained documents from representatives of the pilot program to determine how the vaccination program was conceived and organized. Documents obtained included vaccination schedules, budgets, the cost of vaccines, cold chain protocol, vaccination procedures, results of a census of children, and records of vaccination, including demographics of children and the dates that vaccines were administered.

### Analysis

Vaccine records were modified to convert from logbooks (Appendix 1) and the MS Excel workbook used before the coup. These records were entered into a database structure that retained necessary variables from the original reports and added more variables for the analyses described in outcomes. Descriptive statistics were used to determine the vaccination status and characteristics of participating children.

### Outcomes

While not pre-specified, the primary outcome closest to the pilot program’s intent was the proportion of children who became up to date for each vaccine. Secondary outcomes were (1) the proportion of children predicted to become up-to-date using inverse survival analysis, (2) the proportion who became up-to-date for combinations of vaccines, (3) the relationship between receipt of vaccines and gender, residence (internally displaced or a local village), being in the prespecified target population (census), and (4) adherence with international standards for the evaluation of vaccination programs (World Health Organization’s Health Facility Level Questionnaire) [31].

To determine the proportion of children who became up to date for individual vaccines, we limited the analysis to zero-dose children who were old enough to receive specific recommended vaccine doses during the five sessions. For example, zero-dose children not yet two months old in May were excluded because they could not receive all three doses of Penta or OPV during the remaining session of the pilot. When a child attended a vaccination session but was not vaccinated, we consulted the health workers to determine the reason: the child was previously vaccinated, too old, sick, or the vaccine was out of stock.

Because children presented for vaccination at different points in time, we used inverse Kaplan-Meier survival analysis [32]. Without censoring to determine the probability of being fully vaccinated for each vaccine type by month since the first participation for zero-dose children. To calculate probabilities, the population under consideration for each vaccine type only included children who were age-eligible and – based on their first date- could receive all recommended doses by the end of the program. For the Penta and Polio population, the constraints were participation in at least one of the March, April, and May sessions and at least two months old in May. To be age-eligible for completion of the MMR series, children had to participate in the March, April, May, or June sessions and be at least nine months old in June. Finally, the BCG eligibility was a maximum age of 12 months during the first participation. The data was analyzed utilizing Pandas version 1.4.4 in the Python programming language.

## Results

### Conception, setting, and financing

**Fig. 1:**
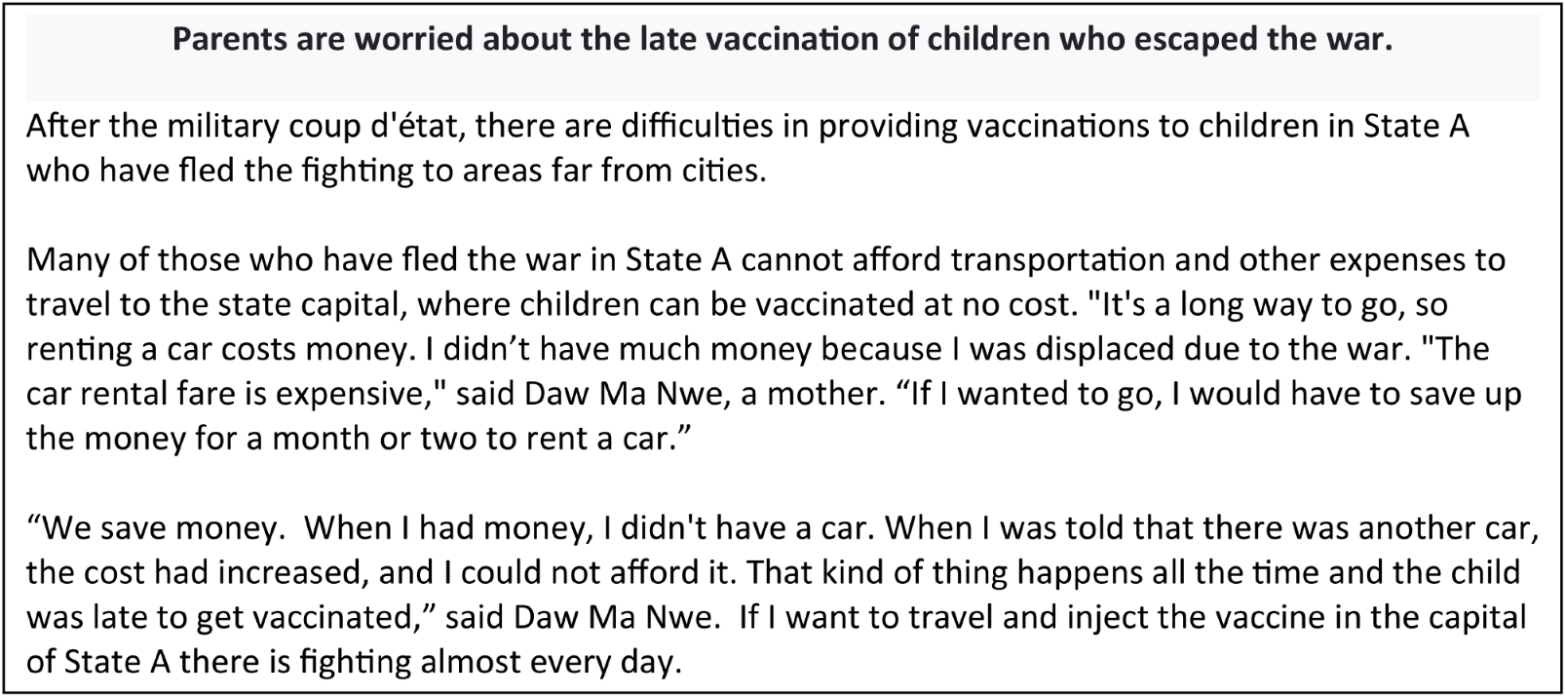
Edited translation of a local news article on mothers’ difficulty in accessing vaccinations for their children

The program was conceived in 2022 and 2023 in response to queries from parents who were aware of the importance of childhood vaccination programs and worried about the lack of one in their state (Fig. 1). The parents urged local ethnic health workers to vaccinate their children.

In 2022, the Team developed a proposal to vaccinate the 6,986-childbirth cohort of their State with the 11 vaccines recommended in Myanmar (Table 1). They approached UNICEF, WHO, and GAVI for financial assistance in obtaining and transporting vaccines. However, only modest technical support (e.g., UNICEF’s advice on cold chain and inspected cold-storage facilities where the vaccines were stored) could be provided (source: authors notes, personal communications, and email correspondence available on request). Instead, the team was urged to conduct a pilot study.

**Table 1:** Vaccines previously used in Myanmar’s routine vaccination program and those used in the pilot

			Cost per dose		Doses per vial	
Vaccine	Routine vaccination schedule	pilot study schedule	pilot	GAVI	pilot	GAVI
BCG	Birth to 2 months (eligible up to 1 year of age)	Same	$2.35	$0.14	20	20
Hepatitis B	Birth dose	Not included	–	$0.49	–	1
Penta	One dose at ages 2, 4, and 6 months	One dose monthly for three months between 2 months and three years (Td after that)	$15.88	$1.24	1	1
OPV	One dose at ages 2, 4, and 6 months	One dose monthly for three months starting at 6 weeks through at any ages	$1.62	$0.18	10	10
Injectable polio	One dose at age four months	Not included	–	$2.80	–	1
MMR	One dose at ages 9 and 18 months	One dose monthly for two months starting between 9 months and 15 years	$10.29	$3.56	1	1
Pneumococcal conjugate	One dose at ages 2, 4, and 6 months	Not included	–	$2.90–$4.00	–	5
Japanese encephalitis	One dose at age nine months	Not included	–	$0.45	–	5
Rotavirus	One dose at ages 2 and 4 months	Not included	–	$2.00	–	5
HPV	One dose at age 9 and 10 years (girls)	Not included*	–	$4.50	–	1
Td	During pregnancy, one dose at first contact and four weeks later	During pregnancy, one dose at first contact and four weeks later	$0.47	$0.12	10	10

*Note* BCG, bacillus Calmette-Guérin; Penta, Diphtheria pertussis, tetanus, hepatitis B, Haemophilus influenza type B; OPV, Oral polio; MMR, Measles, mumps, rubella; HPV, Human papillomavirus; Td, Tetanus, and diphtheria; Not included because funds were insufficient to purchase; When possible, the vial size in the UNICEF catalog most similar to the vial size of vaccines purchased in the neighboring country was used

### Setting

In early 2023, one Team member raised funds through private donations (primarily from Myanmar expatriates in the United States and the United Kingdom). When funding was secured, the other team members chose a setting, Village A and surrounding IDP camps in the state. Village A had a 16-bed hospital and, before the coup, had a peaceful and pastoral population with low health literacy. After the coup, the military junta cut off virtually all transit of vaccines as well as all other humanitarian aid from international non-governmental organizations and United Nations agencies [8]. IDPs migrated to Village A because it was believed to be secure. Most IDPs live in bamboo huts with tarpaulin walls and roofs (Appendix 2).

### Acquisition of vaccines

In early March 2023, Team members traveled to a neighboring country and purchased cold storage boxes pre-qualified by WHO. With the assistance of the local government officials in the neighboring country, vaccines manufactured by the Serum Institute of India (the same manufacturer that produces vaccines for GAVI/UNICEF) were sought at a private pharmacy. However, the Team found that the funds collectively were only sufficient to purchase five of the 11 vaccines because retail prices in the neighboring country were 550% greater than the charge offered by GAVI (Table 1). The vaccines were transferred to a cold storage room closer to the border. While maintaining the cold chain, the vaccines were smuggled into Myanmar. With help from friendly resistance fighters, transit was along jungle backroads to avoid the military junta’s checkpoints. The vaccines arrived in 13.5 h, their temperature monitors were checked, and they were transferred to solar-powered refrigerators.

### Vaccination sessions

All aspects of the vaccination sessions were supervised by a Team of Civil Disobedience Movements (described in the Contributors section), who had mid-level positions in the pre-coup MOH. All but one had responsibilities for vaccination programs. In February 2023, the Team and nurses in Village A and neighboring IDP camps worked with local leaders to obtain a list of their residents. The nurses then contacted parents and constructed a census of 153 children under the age of 1 year. Parents in the village and IDP camps were alerted about the date of the vaccination sessions. On the day of each vaccination session, parents received an orientation session that included information about the diseases that vaccines prevent, the benefits of getting vaccines, and possible side effects after vaccination, including side effects that required a return visit. One of us (Dr. Alfred) examined each child to ensure no contraindications to vaccination. When mothers brought children to the immunization session, vaccinators asked the parents if the child had received any previous vaccines, determined which vaccines were needed, and entered the vaccinations received on a handwritten line-list registry (Appendix 1).

**Table 2:** Characteristics of children participating or not participating in the pilot

	In the census, did not participate in vaccination sessions	In the census, participated in vaccination sessions	Not in census, participated in vaccination sessions	Total participated in vaccination sessions
All children	69	84	100	184
** Sex**				
Female	45 (54%)	39 (46%)	54 (54%)	93 (51%)
Male	24 (46%)	45 (54%)	46 (46%)	91 (49%)
Age, median, months	6.4 (IQR 6.0)	7.5 (IQR 6.8)	5.6 (IQR 7.3)	6.3 (IQR 7.1)
**Residence**				
IDP camp	56 (81%)	33 (39%)	46 (46%)	79 (43%)
Village	13 (19%)	51 (61%)	54 (54%)	105 (57%)
**Vaccination status before the pilot**				
Zero-dose	unknown	74 (88%)	71 (71%)	145 (79%)
Partially vaccinated	unknown	10 (12%)	29 (29%)	39 (21%)

### Participants

Eighty-four (55%) of the children in the census participated in the pilot. A greater proportion of census participants were from villages (61%) than IDP camps (39%) (Table 2). Other characteristics of children who participated and those who did not participate were similar.

**Fig. 2:**
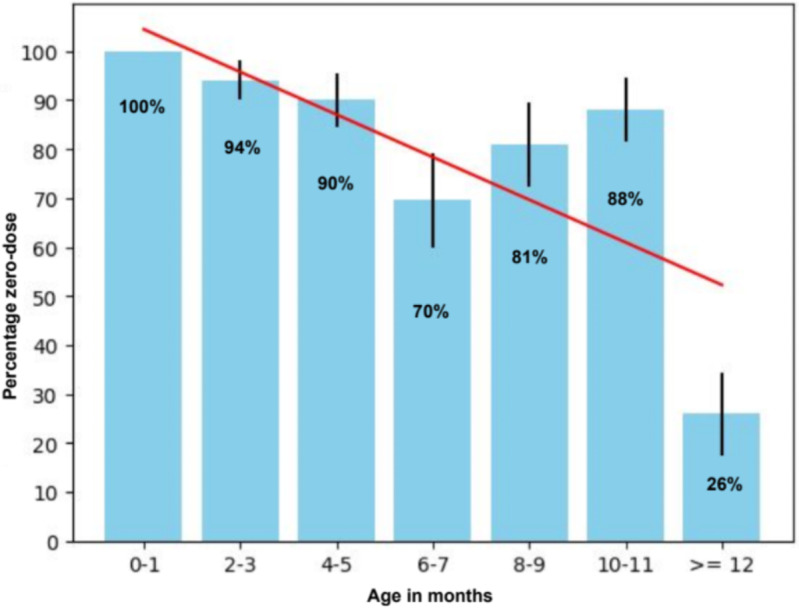
Percentage of zero-dose (no previous vaccinations) children by age group

Since fewer children attended vaccination sessions than expected and families not in the census requested their children be allowed to participate, the Team allowed 100 additional children to participate. Of the 184 children who ultimately participated, 145 (79%, median age five months [IQR 6.5]) were zero-dose, and DPT-1 coverage was 18%. The proportion of zero-dose children was inversely related to age (Fig. 2).

### Participation and outcomes

After the first month, the number of new participants (both zero-dose and incompletely vaccinated) remained steady, averaging 17 new per month (Appendix 3). The number of returning participants remained steady until June but fell in July when most previous participants had completed their vaccine series.

**Fig. 3:**
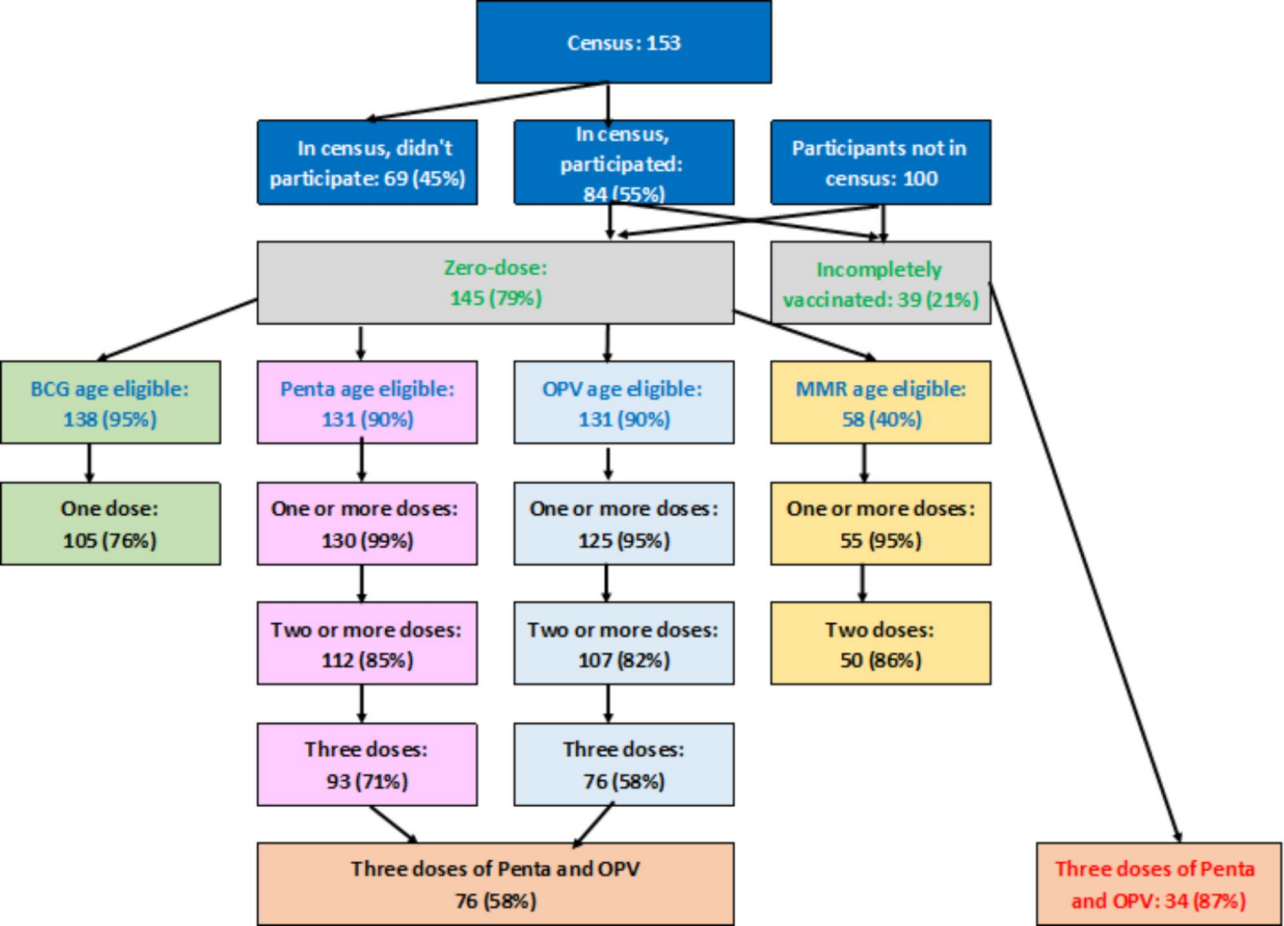
The flow of children presenting for vaccination. Those who were/were not in the census (white text, blue background); zero-dose or incompletely vaccinated (green text, grey background); zero-dose children who were age-eligible (blue text, green background); total number and proportion of zero-dose age-eligible children vaccinated (black text); previously vaccinated children are in red. BCG: bacillus Calmette-Guérin; Penta: Diphtheria pertussis, tetanus, hepatitis B, Haemophilus influenza type B; OPV: Oral polio; MMR: Measles, mumps, rubella

76% of age-eligible zero-dose children received one dose of BCG, 71% three doses of Penta, 58% three doses of OPV, and 86% two doses of MMR (Fig. 3). The recommended doses of BCG, Penta, and OPV were received by 56%. Of 51 children eligible for all four vaccines, 36 (71%) received them. Polio vaccine series completion was limited by stockout when the vaccines were initially purchased, delaying the first doses by a month. BCG vaccine administration was limited by stockout at the vaccination site halfway through the May session.

The Village or IDP camp leaders contacted the families of children who did not participate, asked why they did not participate, and were reminded about the next vaccination session. Demand for vaccination continued through July, but vaccines could not be restocked due to monsoon-flooded roads and the military junta’s blockade of traditional supply routes.

There were no differences between the proportions of male/female or IDP camps/village residents who received complete courses of each vaccine. Of the 39 partially vaccinated children, 37 (95%) received BCG, 37 (95%) received three doses of Penta, 34 (87%) received three doses of OPV, and 27 (69%) received two doses of MMR.

**Fig. 4:**
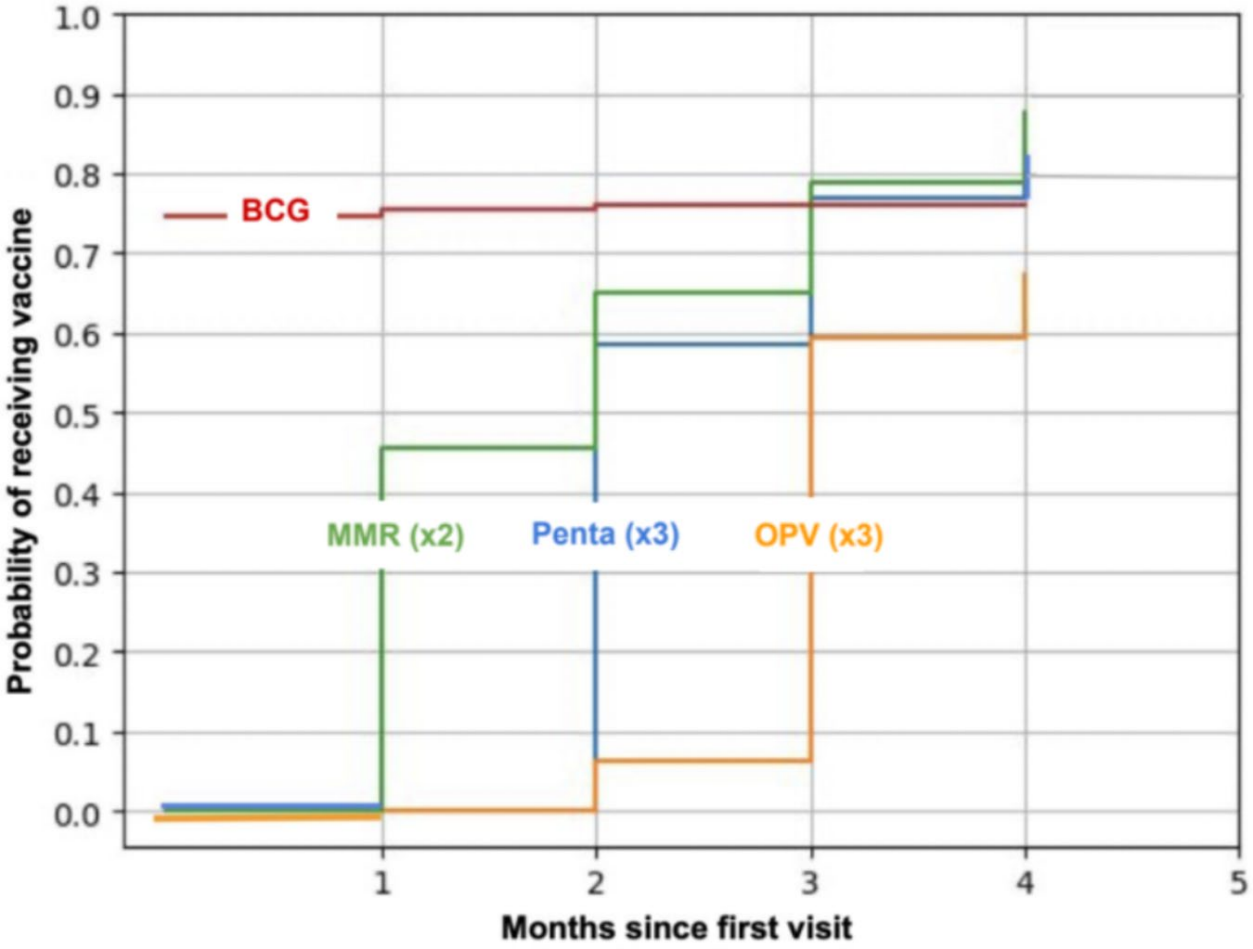
Inverse survival probabilities for zero-dose age-eligible children receiving recommended doses of each vaccine. BCG, bacillus Calmette-Guérin; Penta, Diphtheria pertussis, tetanus, hepatitis B, Haemophilus influenza type B; OPV, Oral polio; MMR, Measles, mumps, rubella

Using inverse survival analysis, the probability of age-eligible children receiving two doses of MMR by the end of the program was 88% (95% confidence interval [CI] 79–96%), three doses of Penta 82% (95% CI 75%–89%), one dose of BCG 76% (95% CI 69%–83%), and three doses of OPV 67% (95% CI 59%–76%) (Fig. 4). OPV stockout in March reduced the probability of receiving the third dose of OPV 2 months after the first visit to 6%, compared to 58% for the third dose of Penta. Due to the stockout of BCG in mid-May, the probability of receiving BCG flatlined to 76%. The rate of increase in probabilities decreased over time because of dropouts. The survival probabilities for becoming up to date for each vaccine were greater than the proportion of children who became up to date during the program since the latter considered a wider range of participants, some of whom did not have enough time left to become up to date (these populations attended at least once while the populations for survival analysis had an additional restriction for months of participation).

### Health facility-level questionnaire

The WHO health facility-level questionnaire had not been completed before this evaluation. Based on our questionnaire administration, we found 74% compliance with the indicators (Appendix 4). Areas of weakness included a lack of written (as opposed to verbal) plans, insufficient vaccine supply, a deficient data collection and analysis protocol, and a lack of staff for optimal communication, social mobilization, and vaccine-preventable disease surveillance.

## Discussion

Mothers who expected their children to be vaccinated pressed local health workers to do so. Knowing that neither the military junta nor UN agencies could supply vaccines, the healthcare workers turned to private fundraising. Vaccines and cold-chain supplies were purchased from retail pharmacies and smuggled to Myanmar, and a vaccination program was organized. At the end of the five monthly vaccination sessions, the probability that zero-dose children would receive the recommended doses of the five vaccines was greater than two-thirds. Although the prespecified end date for this evaluation was July 2023, the vaccine demand continued.

Perhaps as important as the coverage, the pilot represented a ray of hope in the state, where vaccines were largely unavailable. The pilot was locally led, did not require working with the military junta, and was not subjected to top-down policies and political infighting that have plagued vaccination programs in ethnic minority states [33]. It provided much-needed refresher training to Civil Disobedience Movement health personnel in the state, without whom vaccination could never be resumed. Finally, the pilot led to donor funding for expansion to 2,000 children in the state and programs in other states of Myanmar.

### How does childhood vaccine coverage in Myanmar compare with other countries?

After the coup, DPT-1 coverage in Myanmar fell to 45%, one of the lowest levels among larger countries globally, similar only to North Korea’s [34]. We are therefore skeptical of the rebound to 81% DPT-1 coverage reported to UNICEF by the military junta in 2022 [35]. Even if correct, this level is lower than that reported in Myanmar any year since 2003 [5]. However, our finding that – in early 2023, only 18% of children in the pilot had received DPT-1 – leaves no doubt about the detrimental effects of the military junta’s embargo of vaccines on childhood vaccination [35, 36]. Without an independent population-based survey, the actual national childhood vaccination coverage can only be speculated upon; our belief that the problem is more widespread is supported by a recent report that - at the beginning of a similar campaign in another state in Myanmar, only 5% of 273 children (median age eight months) had received DPT-1 (data available on request).

With WHO [37], UNICEF [38], and GAVI [39, 40] prioritizing vaccination of zero-dose children, why have children in Myanmar fallen through the cracks? Why have United Nations agencies not been able to provide vaccines or financial support? The primary responsibility rests with the junta’s unwillingness to allow the distribution of humanitarian relief, including vaccines, to populations that do not support the junta. Numerous other factors related to the military junta’s actions contributed to the fall in immunization coverage. The number of internally displaced people surged to 1.5 million in 2022 [41]. Many areas in Myanmar – including where this pilot was conducted - rank as among the most hard-to-reach displaced populations in the world [41, 42]. Myanmar’s previous special outreach programs for remote areas, those affected by armed conflict, and those under non-government control [43] have ceased. Finally, our inability to obtain assistance in procuring vaccines in the neighboring country reflects the political pressure they are under to cooperate with the junta [44].

### Limitations of the evaluation and the pilot

Both the evaluation and the pilot had many limitations. Communication within the Team was limited by some of the evaluators’ lack of Myanmar language abilities. Electronic communication in most of the state is intermittent at best as the junta cut off all their electricity, making communication among the evaluation team a constant challenge. Members of the evaluation team in Myanmar have regular direct patient care responsibilities. This prevented the collection of qualitative data from nurses not on the evaluation team, as well as parents and community leaders.

The pilot had numerous methodological weaknesses. When it was conceived, the Team was unaware of the standards in the WHO publication “Vaccination in Acute Humanitarian Emergencies: Implementation Guide,” [45] standards that advised against childhood vaccination in areas such as the state with conflict and unstable vaccine supply. The use of vaccines that cost five times more than those purchased through UNICEF, the inability to obtain the other six recommended vaccines, the hazards of smuggling, and the expenses of overland transportation all decreased the well-documented cost-effectiveness of vaccination [46, 47] and could easily have led to labeling the pilot as unjustifiable. The target of 150 children was small, considering what we believe are millions of zero-dose children in the rest of the country. The loss of children of IDP families to follow-up is another example of the difficulty in reaching lower-income households [6]. Conducting a census just before vaccination initiation would have eliminated children whose families move away before vaccination from those lost to follow up. The data entry protocol used, modified from one developed by the pre-coup MOH, was in part unsuitable without time-consuming cleaning to perform analyses needed to evaluate the pilot optimally. Finally, the lack of written documents on planning pre-specification of primary outcomes prevented valid calculation of statistical significance.

### International actors are not without responsibility

Apart from the junta’s blockage of humanitarian relief, the main obstacle was UN agencies’ lack of support for cross-border vaccination. Our criticism of United Nations agencies is supported by an independent group of former UN mandate holders in a recent report entitled “How the UN Is Failing Myanmar,” which criticizes the UN’s inaction on areas within its purview [48]. They highlight the UN Country Team’s pursuit of appeasing the military junta despite growing risks and ever-fewer results. Action on their recommendation, such as resuming the delivery of humanitarian assistance directly to local humanitarian actors, would alleviate problems that have led to low vaccination coverage.

### Possible steps forward

Some support from public sector donors for local health activities in the state has begun. The success of this vaccination program led a donor to allocate funding for vaccination of 2000 children in 2024. Finally, WHO, UNICEF, and GAVI could reexamine their policy that skews partner investments toward working with partner governments rather than supporting health systems and civil society organizations [42]. The neighboring country’s government, where the Team purchased vaccines, could work with UNICEF to support the acquisition of lower-priced vaccines [43].

The results of this pilot should be considered in future revisions of policies on vaccination in humanitarian emergencies [44]. Regardless of its effect on policy, there can be little doubt that vaccination in conflict settings demands greater investment in health system support and civil partnerships, as Grundy and Biggs’s stated in their 2019 article [42, 43] An international technical advisory group is needed to help improve the humanitarian health and nutrition response for conflict-affected women [49]. The possibility of jointly funding ethnic health civil society and military junta is appealing [50] but probably unrealistic given the level of hostilities.

## Conclusions

Despite many challenges, the Team organized a program that designed, imported, and administered five recommended vaccines to more than 2/3 of participating children. Most of the children were zero-dose, and almost half were internally displaced. While the pilot succeeded as a proof of concept, re-starting vaccination programs for children in the many parts of Myanmar where vaccines are being embargoed will require reducing the dangers and costs of this pilot. Outcome indicators must be established in advance to conduct a more robust evaluation. Additional financial support is necessary to provide/support salaries, training, and tranportation for a stable supply of vaccines and medicines.

The military junta’s blockade of transportation, food, and medical supplies remains the major barrier in the state and many other parts of Myanmar [22]. A sub-national approach that empowers local organizations and their health systems, such as those that led the pilot, is essential and may be the only way to address the continued obstacles in Myanmar’s conflict areas [12, 42, 48]. Such partnerships can help bridge gaps in healthcare delivery and provide valuable local insights, such as those provided in this report. However, even with more support, addressing the problem will not be short-term [33]. Widayat concluded that, despite the enormous amount of funds provided by international donors between the democratic elections in 2016 and the coup, the assistance was, at best, temporary and, at worst, a placebo [51].

### Electronic supplementary material

Below is the link to the electronic supplementary material.

Supplementary Material 1

Supplementary Material 2

Supplementary Material 3

Supplementary Material 4

## Data Availability

Data is provided within the manuscript or supplementary information files are available upon request to Dr. Daniel Fishbein, dbf1dbf1@gmail.com.
