# Supplementary material for: Struggling to resume childhood vaccination during war in Myanmar: evaluation of a pilot program
Source: Int J Equity Health. 2024 Jun 13;23:121. doi: 10.1186/s12939-024-02165-9 (PMC11177543; doi:10.1186/s12939-024-02165-9)
Supplement: Supplementary file 1 — Supplementary Material 1 [file 12939_2024_2165_MOESM1_ESM.docx]

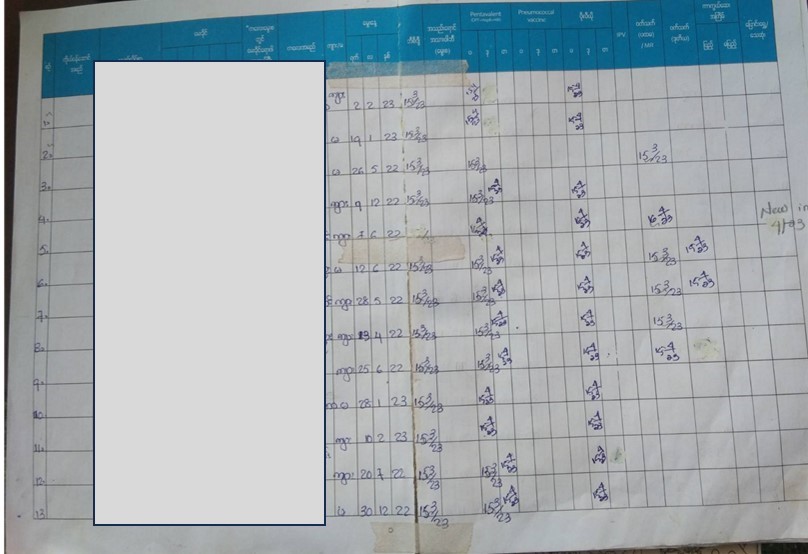


Appendix 1. Photograph of the logbook, the original source of data, recording which vaccines were administered. Data is from March and April. The grey rectangle hides identifying information. Columns to the right of the grey rectangle are sex, date of birth, date of BCG, PENTA, pneumococcal, OPV, Japanese B, MMR, and notes. The first two participants received BCG and the first dose of PENTA in March, and since OPV was out of stock, it was not received until April. The third participant received BCG, OPV, and Penta in March but appeared not to return in April.
