# Supplementary material for: Struggling to resume childhood vaccination during war in Myanmar: evaluation of a pilot program
Source: Int J Equity Health. 2024 Jun 13;23:121. doi: 10.1186/s12939-024-02165-9 (PMC11177543; doi:10.1186/s12939-024-02165-9)
Supplement: Supplementary file 2 — Supplementary Material 2 [file 12939_2024_2165_MOESM2_ESM.docx]

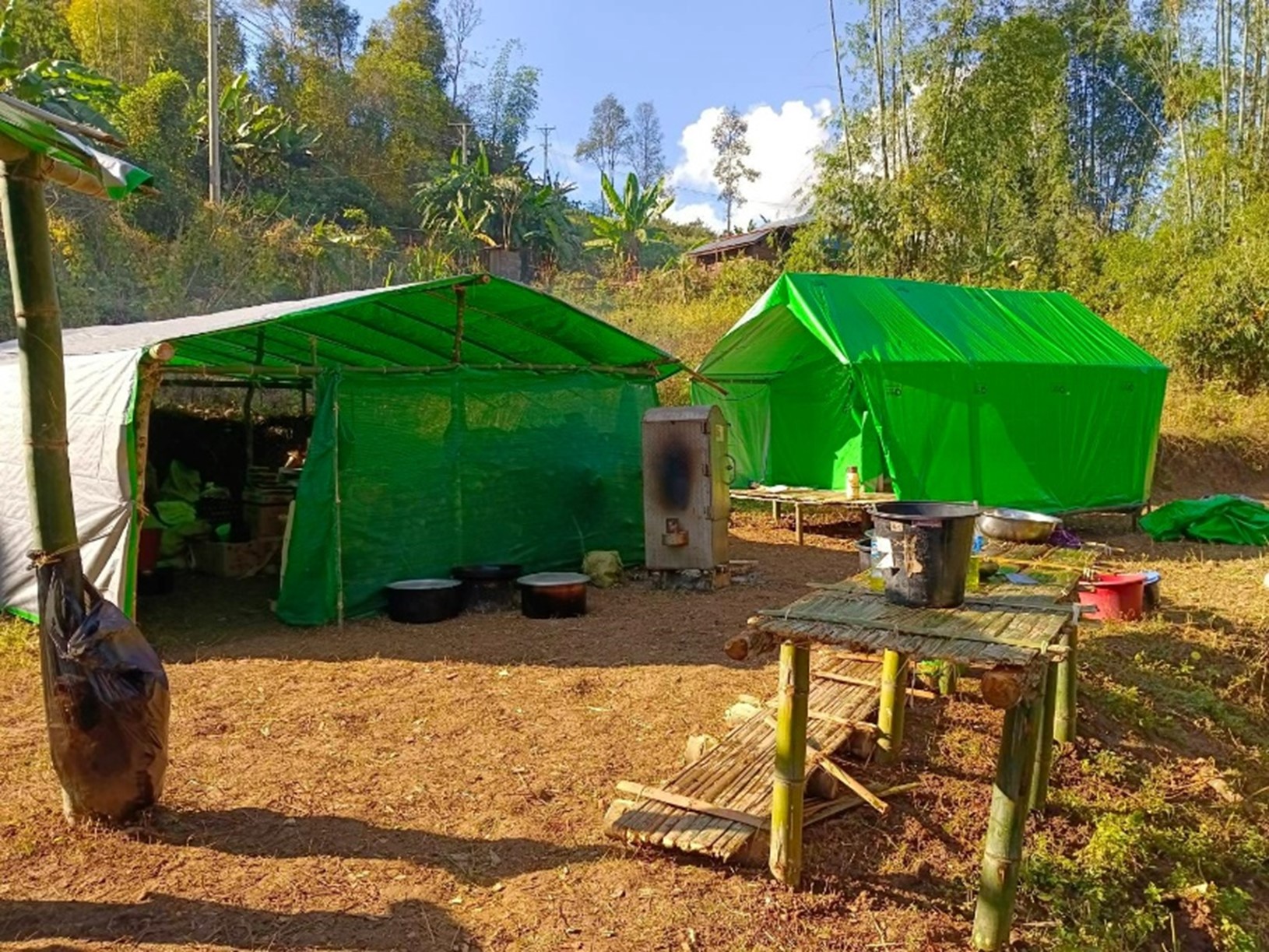


**Legend**. The floors are made of woven bamboo. Two or three families live in a single hut. Water supply is from nearby streams or springs, and sanitation is in an open space. There are no latrines or pit latrines built of bamboo.
