# Supplementary material for: Struggling to resume childhood vaccination during war in Myanmar: evaluation of a pilot program
Source: Int J Equity Health. 2024 Jun 13;23:121. doi: 10.1186/s12939-024-02165-9 (PMC11177543; doi:10.1186/s12939-024-02165-9)
Supplement: Supplementary file 3 — Supplementary Material 3 [file 12939_2024_2165_MOESM3_ESM.docx]

| **Appendix 3.** The number and proportion of new, returning, and total participants per month, divided into zero-dose and incompletely vaccinated. | | | | | | | | | | | |  |
| --- | --- | --- | --- | --- | --- | --- | --- | --- | --- | --- | --- | --- |
|  | **New participants** | | | | **Returning participants** | | | | **All participants** | | |  |
| **Session month** | Zero dose  before Pilot | Incompletely  vaccinated  before Pilot | **Total** | Zero dose  before Pilot | | Incompletely  vaccinated  before Pilot | **Total** | Zero dose  before Pilot | | Incompletely  Vaccinated  before Pilot | **Total** | |
| March, 2023 | 100 (87%) | 15 (13%) | **115** | -- | | -- | -- | 100 (87%) | | 15 (13%) | **115** | |
| April, 2023 | 10 (71%) | 4 (29%) | **14** | 86 (89%) | | 11 (11%) | **97** | 96 (86%) | | 15 (14%) | **111** | |
| May, 2023 | 6 (43%) | 8 (57%) | **14** | 74 (84%) | | 14 (16%) | **88** | 70 (78%) | | 20 (22%) | **90** | |
| June, 2023 | 14 (67%) | 7 (33%) | **21** | 96 (89%) | | 12 (11%) | **108** | 110 (85%) | | 19 (15%) | **129** | |
| July, 2023 | 15 (75%) | 5 (25%) | **20** | 37 (84%) | | 7 (16%) | **44** | 52 (81%) | | 12 (19%) | **64** | |
| **All** | **145 (79%)** | **39 (21%)** | **184** | **293 (87%)** | | **44 (13%)** | **337** | **428 (84%)** | | **81 (16%)** | **509** | |
