# Supplementary material for: Struggling to resume childhood vaccination during war in Myanmar: evaluation of a pilot program
Source: Int J Equity Health. 2024 Jun 13;23:121. doi: 10.1186/s12939-024-02165-9 (PMC11177543; doi:10.1186/s12939-024-02165-9)
Supplement: Supplementary file 4 — Supplementary Material 4 [file 12939_2024_2165_MOESM4_ESM.docx]

**Appendix 4. WHO** Health Facility Level Questionnaire completed as part of the evaluation **.**

**FORM 3. HEALTH FACILITY LEVEL QUESTIONNAIRE**

***INSTRUCTIONS.*** *This questionnaire is organized by topic area. It is meant to guide your interview and collect a few standard indicators. Note that the most important part of collecting data is engaging the interviewees, listening, and asking probing questions to get a complete picture. Request hardcopy documents when indicated. Make sure all questions are answered and with one response per question unless otherwise indicated. Use the back of the sheet to make notes or separately in notebook. The green annotations provide discussion points; notes from these questions do not need to be entered in a database but can be presented during debriefing.*

**Interviewer name: CYNTHIE TIN-OO and DAN FISHBEIN . Date of interview (DD/MM/YY): 7 - 12 SEPTEMBER 2023**

| **Name** |  |
| --- | --- |
| District name |  |
| Health Facility name | STATION HOSPITAL |
| Health Facility Type | **X** Hospital ☐ Clinic ☐ Health Post |
| Health Facility Ownership | X Government ☐ Private |
| Setting | ☐ Urban ☐ Semi-urban X Rural |
| Staff interviewed | ☐ Health facility in-charge **X** Immunization Focal Point ☐ Vaccinator X Cold chain focal point ☐ Surveillance focal point ☐ Other staff |

*For each person participating in the interview, list their name, role, and organization on a new line.*

| **Name** | **Organization** | **Role/Position** |
| --- | --- | --- |
| APRIL |  | TEAM LEADER |
| DR. ALFRED |  | PHYSICIAN |
| EMILY |  | NURSE |
| HEIN |  | HEALTH ASSISTANT |
|  |  |  |

| PROGRAM MANAGEMENT and FINANCING*Discuss immunization planning, budgeting, and funding. Note significant strengths and weaknesses.* | | |
| --- | --- | --- |
|  | Does the HF have an immunization field guide or job aide? *Note what needs or improving:* | ◻ Yes – good guide  ◻ Partial – needs updating, improving  ✠ No  ◻ Don't know/not seen |
|  | Does the field guide provide clear instruction sufficiently covering all aspects of vaccine management and service delivery – including new vaccines? *Is guide clear, missing any components.* | ◻ Sufficient  ◻ Somewhat sufficient  ◻ Insufficient  □ Don't Know  X Not applicable (no guide) |
|  | Does HF have an up-to-date and clear immunization schedule.*Request a copy This may be in a field guide or somewhere else. Check to see if staff have any questions.* | **✠** Yes  ◻ No  □ Don't Know |
|  | Does the HF have a high quality and up-to-date micro-plan (work plan)?*Request a copy. Note: define 'up-to-date', discuss process of drafting the plan, how often this is done* | ☐ Yes  ✠ Partial  ☐ No  ☐ Don't know |
|  | Do you have a budget detailing funds needed to conduct immunization activities?  *Request a copy.* *Note: discuss how budget it made, if not – would it be helpful to have a budget etc…* | ✠ Yes – all covered  ☐ Partial – some covered  ☐ No  ☐ Don't know |
|  | **Do funds received (government and donor) sufficiently cover all immunization activities including to reach hard-to-reach populations?**  *Note key underfunded areas.* | ◻ Sufficient  ◻ Partially sufficient  ✠ Insufficient  □ Don't Know |
|  | **In the past 12months, were any critical immunization program activities cancelled because of lack of funds?**  *Note types of activities cancelled* | ✠ Yes  ☐ No  ☐ Do not know |
|  | Does the local government support immunization activities? *Check all that apply.* *Note type of support* | ◻ Financial  ◻ Advocacy/Demand generation  ◻ Service Delivery  □ Other Role  ✠ No Role |
|  | **What role do any non-government organizations provide for local immunization activities? *Check all that apply.***  *Note: Names of NGOs; types of support*  ***Civil Society Ethnic Health Organizations led Pilot program*** | ✠ Financial  ✠ Advocacy/Demand generation  ✠ Service Delivery  □ Other Role  □ No Role |
|  | **What role(s) does the community have with immunization services? *Check all that apply.***  *Note if organized community group mechanism for engagement.*  ***Community leaders informed parents about immunization at the meetings, mobilized parents to attend the fixed site sessions and helped nurses to track the defaulter*** | ☐ Advocates for immunization  ✠ Support in fixed site sessions  ☐ Support in outreach sessions  ✠ Help with defaulter tracking.  ✠ Discuss immunization performance at community meetings.  ☐ Don't Know  □ No Role |
| Note any further comments about this section. | | |

| HUMAN RESOURCE MANAGEMENT*Discuss HF-level human resource for immunization, needs for capacity building.* | | |
| --- | --- | --- |
|  | Are any of these aspects of immunization services not sufficiently covered due to lack of staff time or capacity?  *Tick all that apply.* | ☐ Microplanning☐ Fixed site vaccination✠ Outreach vaccination ✠ Defaulter tracking & vaccination ☐ Cold Chain maintenance☐ Vaccine stock, reporting data✠Analysing data, using data☐ Community engagement☐ All aspects sufficiently covered |
|  | Does the HF have any specific training needs?  *Tick all that apply.* *Note 'other' trainings needs if mentioned.* | ◻ Microplanning  ◻ Immunization practice/new vaccines ◻ Immunization Practice  ◻ Optimizing Service Delivery  ◻ Cold chain, Vaccine management  ✠ Data management & use  ◻ Communication/Demand  ✠ Surveillance/AEFI  ◻ Other  ◻ No training is needed |
|  | In the last 6 months, how many times has this HF received an immunization-related supervisory visit? | ✠ No visits  ☐ 1 visit  ☐ More than 1 visit  ☐Don't know |
|  | Is there any observed evidence of supervisory visits to the HF that have been received in the last 6 months? | ☐ Yes - Written feedback in last 6 months  ☐ Partial - Signature only  ✠ No evidence in last 6 months |
| Note any further comments about this section. | | |

| VACCINE SUPPLY, QUALITY, LOGISTICS*Discuss immunization supply chain system in general including human resource capacity to support the system. Note major strengths and weaknesses.* | | |
| --- | --- | --- |
|  | **Does the HF have a person responsible for vaccine supply and management in place and adequately trained?**  *Reflect back on the answers given in the previous HR section; confirm if this person is trained and in place.* | ✠ Yes, in place and trained.  ◻ Partial, in place but not adequately trained.  ◻ No  ◻ Don't know. |
|  | **Is the HF cold storage sufficient (function, space)?**  *If not sufficient, describe the insufficiency:* | ✠ Yes, both space and function  ◻ Partial  ◻ No  ◻ Don't know. |
|  | **Is the HF process of forecasting vaccine needs sufficient?**  *Request the stock management tools, vaccine use and request forms* | ✠ Sufficient  ◻ Partially sufficient  ◻ Insufficient  □ Don't Know |
|  | Are the vaccine stock management tools properly filled in?*Verify* | ✠Yes  ☐ Partially  ☐ No  ☐ Don't Know |
|  | Does the HF have a sufficient way to receive vaccines?*Discuss the process and note any strengths or weakness, possibilities for improvement.* | ✠ Sufficient  ◻ Partially sufficient  ◻ Insufficient  □ Don't Know |
|  | In the past 6 months, has there been a stock out of any vaccine? | ✠ Yes  ◻ No  ◻ Don't know. |
|  | **If there were stockouts, specify which vaccine/supply has been out of stock and for how long**  Vaccine name: **OPV** ✠ **Stock-out less than 2 months** ◻ 2 or more months ◻ Don’t know  Vaccine name: **BCG**_ 🗹 Stock-out less than 2 **months** ✠ **2 or more months** ◻ Don’t know  Vaccine name: _____________ ◻ Stock-out less than 2 months ◻ 2 or more months ◻ Don’t know  Vaccine name: _____________ 🗹 Stock-out less than 2 months ◻ 2 or more months ◻ Don’t know | |
|  | If there were stockouts, what are the reasons? Too many BCG vials had to be opened and discarded | ◻ Higher level stock out  ◻ Inaccurate forecasting  ✠ More wastage than expected  ◻ More demand than expected  ◻ Other (specify):  ◻ Don't know. |
|  | **What waste management method does the HF use for discarding of needles and syringes?** *If other, describe:* | ✠ burn and bury in a pit  ◻ uses incinerator  ◻ district picks up waste  ◻ other |
|  | **Is the current practice of waste management in the district sufficient?** | ◻ Sufficient  ✠ Partially sufficient  ◻ Insufficient  □ Don't Know |
|  | **OBSERVATION. In HF vaccine supply and waste management, do you observe any of the following:** *Tick all that apply*  🗹 This HF does not have a refrigerator  ◻ Refrigerator not working  ◻ Expired vaccine  ◻ VVM inner square is same color or darker than the outer circle, ie, should be discarded – on any vaccine  ◻ Expiration date not written on vials  ◻ Freeze sensitive vaccines frozen or vulnerable to freezing because of placement or temp in the refrigerator  ◻ Fridge tag showing alarm  ◻ Freeze tag showing alarm  ◻ Temp not monitored  ◻ Temp out of range in the past 3 months  ◻ Non-vaccine items stored with vaccines  ◻ Other issue (specify): | |
| Note any further comments about this section. **new gasoline generator is needed** | | |

| SERVICE DELIVERY*Discuss whether immunization services are well performing and reach all target populations. Remember you should also try and observe a vaccination session and complete the corresponding tool.* | | | |
| --- | --- | --- | --- |
|  | Does the HF have a map showing communities in the catchment areas including outreach sites if outreach is conducted? | | ☐ Yes, adequate  ✠ Partial - missing some information  ☐ No ☐ Don't know. |
|  | Are there any geographic areas or communities with many under-vaccinated children? *If Yes, describe reasons, actions to try and improve coverage*  ***The communities constantly moved from one place to another due to conflicts and fighting between military and resistance fighters.*** | | ✠ Yes  ☐ No  ☐ Don't know |
|  | How often does the HF hold fixed-site immunization sessions? | | ◻ 5-7 days per week  ◻ 2-4 days per week  ◻ 1 day per week  **Monthly**  ◻ Has not held fixed-site sessions  ◻ Don't know |
|  | In the last 3 complete months, were any fixed-site immunization session cancelled and for what reasons? *Tick all that apply.* | | ✠ No, no fixed sessions were cancelled  ◻ Partial - lacked staff to conduct session  ◻ Partial - lacking funds/salary  ◻ Partial - lacking vaccines or supplies  ◻ Partial - other – please note reason  ◻ Don't know. |
|  | **What are the main barriers to having high quality fixed site immunization sessions in this HF?**  *Tick all that apply.* | | ◻ No barriers - sessions are high quality  ◻ Long wait times  ◻ Inconvenient schedule for caregivers  ◻ Not enough staff  ✠ Data recording cumbersome  ◻ Frequent stock outs of supplies  ◻ Lack of community volunteers  ◻ Other;  ◻ Don't know. |
|  | Does the HF conduct OUTREACH immunization sessions and if so how often? | | ✠ HF does not conduct outreach  ◻ 1 per week  ◻ 1 per every 2 weeks  ◻ 1 per every 4 weeks  ◻ Less than 1 per every 4 week  ◻ Don't know |
|  | **If this HF conducts OUTREACH,**  *(if no outreach skip this set of questions)* | **are any of the following obstacles for having a good outreach session?** *Tick all that apply* | ◻ No barriers – outreach is high quality  ◻ Insufficient transport  ◻ Not enough staff  ◻ Insufficient reimbursement for staff  ◻ Poor planning (ie. Timing or location)  ◻ Frequent stock outs  ◻ Other (specify):  ◻ Don't Know |
|  |  | **how many outreach sessions were scheduled to take place over the last 3 complete months?** | **None**  ◻ Don't Know |
|  |  | **how many outreach sessions were cancelled during the last 3 complete months?** | ###  ◻ Don't Know |
|  |  | if sessions were cancelled, what were the reasons? | ◻ No outreach sessions were cancelled  ◻ Lacking staff to conduct session  ◻ Lacking transportation  ◻ Lacking funds/salary  ◻ Lacking vaccines or supplies  ◻ Other – please note reason  ◻ Don't know |
|  | **Would HF vaccinate a 22-month old child if they never received any vaccines?**  **all except BCG** | | ◻ Yes - all eligible vaccines  ◻ Partial – only some eligible vaccines  ◻ No  □ Don't Know |
|  | Ask how HF tracks children who have been vaccinated at this HF in the past but are now missing vaccinations (defaulters). Select the option that best describes the response. *If not sufficient – note the problem.* **When the HF find the reason, the staff will try to convince the parents to attend the vaccination session** | | ◻ Sufficient  ✠ Partially sufficient  ◻ Insufficient  □ Don't Know |
|  | Ask how HF reaches children who are missing vaccinations. Select the option that best describes the response. *If not sufficient – note the problem.* **HF contacted mothers through community leaders to track children missing vaccinations.** | | ◻ Sufficient  ✠ Partially sufficient  ◻ Insufficient  □ Not applicable – they don't track  □ Don't Know |
|  | How many children eligible for measles vaccine should be present before opening a measles vaccines vial? | | ✠ if 1 child present, would open vial  ☐ need at least 2-3 children to open vial  ☐ need 4–10 children to open vial  ☐ Don't know |
|  | **Are any immunization services routinely integrated with other health interventions?**  *Discuss which vaccines and health interventions are integrated, advantages and disadvantages to integration, potential for integration* | | ◻ Growth Monitoring  ◻ Vitamin A  ◻ Deworming  ◻ Bednet  ◻ Other  NONE |
| Note any further comments about this section. | | | |

| COVERAGE MONITORING*Discuss district approach to data recording, reporting and use as well as coverage monitoring. Note major strengths and weaknesses.* | | |
| --- | --- | --- |
|  | **Is the person responsible for data management & use` in place and adequately trained?**  *Reflect back on the answers given in the previous HR section; confirm if this person is trained and in place.* | ✠ Yes – in place and adequately trained  ◻ Partial – in place but not adequately trained  ◻ No  ◻ Don't know |
|  | **Is there any confusion or problems with any of the vaccine-related forms?**  *Note any issues – check if there is any confusion around recording vaccines that are given late (after 12m* | ◻ Yes there is some confusion or problems  ✠ No  ◻ Don't know |
|  | **If a child receives DTP3 at 22 months is it clear how this should be recorded and reported on all the forms discussed above?**  *What is not clear? Which forms are not clear?* | ◻ Yes, its clear on all forms  ✠ No – it is not clear on all forms  ◻ Would not administer DPT this late  ◻ Don't know. |
|  | **In the past 12months, has there been a stock out of Government-issued data recording and reporting tools?**  *Tick all that apply. Note any challenges with availability of these documents****.***  ***Because of conflicts and fighting in the region, none of the tools were provided by military junta-controlled Ministry of Health*** | ✠ Tally sheets  ✠ Immunization register  ✠ Monthly report forms  ✠ Monitoring chart  ✠ Vaccine stock book  ◻ Other (specify):  ◻ Don't know |
|  | How do you estimate the number of <1 year olds in your catchment area? *Note if 'OTHER'* | ☐ Birth registry  ✠ Community/local counts  ☐ Receive number from higher authorities  ☐ Other |
|  | Do you feel the official estimates for <1 year olds in your catchment areas accurate? | ☐ Too high  ☐ About accurate  ☐ Too low  ☐ Do not know  **No official estimates** |
|  | How are monthly vaccine usage and coverage reports sent to the next level? | ✠ **Electronically**  ☐ Paper reports are picked up  ☐ Paper reports are delivered  ☐ Do not know |
|  | **In <x calendar year> how many of the 12 monthly vaccine reports were submitted?** *Verify* | **Program only 5 months old**  _____ # reports submitted (of 12)  ◻ Don't know or not available. |
|  | **In <x calendar year> how of the 12 monthly vaccine reports were submitted on-time to the next level?** *Verify* | **Program only 5 months old**  _____# reports on-time reports (of 12)  ◻ Don't know or not available |
|  | Is a vaccination coverage accurately calculated and plotted on monitoring chart in the last 12 months at this HF?*Verify and observe accuracy* | ☐ Yes, and accurate  ☐ Partially - minor problems  ☐ Partially - major problems  ✠ Partially - blank / not updated  ☐ Not used |
|  | Is a DTP1-DTP3 dropout accurately calculated over the last 12 months?*Verify and observe accuracy; note problems* | ☐ Yes, and accurate  ☐ Partially - minor problems  ☐ Partially - major problems  **☐ Partially - blank / not updated**  ☐ Not used |
|  | How does the HF use immunization coverage data or dropout data? | ☐ Respond to low coverage areas  ☐ Advocate for more resources  ☐ Share or announce to community leaders  ✠ Other- **Does not appear to be used except reports to donors** |
|  | Do you receive feedback on your immunization summary forms?*If Yes – describe, can you see an sample?* | ☐ Yes – once a year  **☐ Yes – more than once a year**  ☐ No  ☐Don't know |
| Note any further comments about this section. | | |

| SURVEILLANCE and AEFI monitoring*Discuss surveillance systems and capacity at district level. Note major strengths and weaknesses.* | | |
| --- | --- | --- |
|  | **Is the person responsible for surveillance activities in place and adequately trained?**  *Reflect back on the answers given in the previous HR section; confirm if this person is trained and in place.* | 🗹 Yes, in place and trained  ✠ Partial, in place but needs training  ◻ No, currently not in place  ◻ No, there is no such person responsible  ◻ Don't know |
|  | **Do you have a surveillance field guide or standard operating procedures?** | ☐ Yes  ✠ No  ☐ Don't Know |
|  | Do you work with the community to identify diseases? *Note how this works* | ☐ Yes - good collaboration  ✠ **Partial - potential to improve**  ☐ No  ☐ Don't Know |
|  | **What is the case definition for AFP?**  **A suspected case is defined as a child under 15 years of age presenting with acute flaccid paralysis (including GBS)** | ☐ Correct  ☐ Incorrect |
|  | **What is the case definition for Measles?**  **A suspected case is defined as an illness in a patient with fever and generalized maculopapular rash, or in a patient whom a health care worker suspects has measles** | ☐ Correct  ☐ Incorrect |
|  | **Adverse Event Following Immunization (AEFI**)  **AEFI is defined as any untoward medical occurrence following immunization which does not necessarily have a causal relationship to the vaccine** | ☐ Correct  ☐ Incorrect |
|  | Do you have all the forms and supplies required to investigate and report an AFP or suspected measles case? *If no, what is missing* | ✠ Yes  ☐ No  ☐ Don't Know |
|  | In the past 12months, how often has this HF received written feedback on VPD surveillance results, including lab results? | ◻ Annually  ◻ Quarterly  ◻ Monthly  ◻ Weekly  ◻ A specific times (ie. meetings)  **◻ Never**  ◻ Don't know |
|  | Does HF have written guidance on what to do if an AEFI takes place? | ✠ Yes  ☐ No  ☐ Don't Know |
|  | **What does the HF do if an AEFI takes place?**  **Treat the case first according to guidelines, report to supervisors, refer the case to hospital after adequate treatment, explain the community, continue the immunization session** | ◻ Adequate response  ◻ Partially adequate  ◻ No response  ◻ Don't know |
| Note any further comments about this section. | | |

| DEMAND GENERATION *Discuss activities and plans related to communication/social mobilization on immunization. Note major strengths and weaknesses* | | |
| --- | --- | --- |
|  | **Does the HF have a person responsible for communication & social mobilization who is in place and adequately trained?**  *Reflect back on the answers given in the previous HR section; confirm if this person is trained and in place.* | ◻ Yes, in place and trained  ◻ Partial - in place but needs training  ✠ No, currently not in place  ◻ No, there is no such person responsible  ◻ Don't know |
|  | **Are there significant instances of individual or community-level resistance to vaccination in this district?**  *If yes, please describe groups, concerns, approaches to improve.* | ◻ Yes  ✠ **No**  ◻ Don't know |
|  | What kind of communication & social mobilization activities for immunization does this HF conduct? | ✠ **Through community health workers**  ✠ **Through community groups**  ☐ Using public announcements  ☐ Door to door visits  ☐ Religious leaders  ☐ Social media / Internet / email  ☐ Phone-based messages  ☐ Radio / TV  ☐ Other (specify): |
|  | What are the main information needs from the community for immunization? | ☐ Vaccine safety / AEFI  ☐ Benefits of immunization  ☐ Diseases / specific vaccines  X Vaccine schedule / session times and locations  ☐ Other (specify): |
|  | **Does the HF coordinate with local authorities, community leaders and local partners (ie. religious groups) to support advocacy for immunization?** *Describe how and when local authorities and community leaders are engaged about immunization?*  **Before the immunization began, HF contacted the community leaders to advocate about the immunization, collect the list of children in the communities, and arrange transportation of mothers with children to the immunization sessions. Through the community leaders, HF staff mobilize and educate the parents regarding immunization.** | ✠ Yes – to a good degree  ◻ Partially  ◻ No  ◻ Don't know |

| INTERVIEW IS COMPLETE – Thank you to the interviewees!! |
| --- |

| SUMMARY. *This is a summary for the Reviewer to complete based on overall impressions. This can be completed at the end of the day.* | | |
| --- | --- | --- |
| **How would you (the reviewer) rate the overall capacity of the services in the following areas** | | |
|  | **EPI program management** | ◻ Sufficient ✠ Partially sufficient ◻ Insufficient ◻ Don’t know |
|  | **Human resource management** | ◻ Sufficient ◻ Partially sufficient ✠ Insufficient ◻ Don’t know |
|  | **Vaccine supply and logistics** | ◻ Sufficient ◻ Partially sufficient ✠ Insufficient ◻ Don’t know |
|  | **Delivery of immunization services** | ◻ Sufficient ◻ Partially sufficient ✠ Insufficient ◻ Don’t know |
|  | **Coverage monitoring** | ◻ Sufficient ◻ Partially sufficient ✠ Insufficient ◻ Don’t know |
|  | **Surveillance of VPDs and AEFIs** | ◻ Sufficient ✠ Partially sufficient ◻ Insufficient ◻ Don’t know |
|  | **Demand generation** | ✠ Sufficient ◻ Partially sufficient ◻ Insufficient ◻ Don’t know |
| **What are the main reasons for under-vaccinated children in the HF catchment area?**  **Lack of vaccines** | | |
| **Have you identified best practices or innovations in the HF to share?**  No | | |
| **Main suggestions for improving immunization services in this HF:**  **More vaccines** | | |
